# Are lipid ratios and triglyceride-glucose index associated with critical care outcomes in COVID-19 patients?

**DOI:** 10.1371/journal.pone.0272000

**Published:** 2022-08-01

**Authors:** Marzieh Rohani-Rasaf, Kosar Mirjalili, Akram Vatannejad, Maryam Teimouri

**Affiliations:** 1 Department of Epidemiology, School of Public Health, Shahroud University of Medical Sciences, Shahroud, Iran; 2 Student Research Committee, School of Medicine, Shahroud University of Medical Sciences, Shahroud, Iran; 3 Department of Comparative Biosciences, Faculty of Veterinary Medicine, University of Tehran, Iran; 4 Department of Clinical Biochemistry, School of Allied Medical Science, Shahroud University of Medical Sciences, Shahroud, Iran; Temple University School of Medicine, UNITED STATES

## Abstract

Lipid ratios and the triglyceride and glucose index (TyG) could be a simple biochemical marker of insulin resistance (IR). The current study was carried out to examine the correlation between triglyceride to high-density lipoprotein-cholesterol (TG/HDL-C), total cholesterol to HDL-C (TC/HDL-C), low-density lipoprotein-cholesterol to HDL-C ratio (LDL-C/HDL-C), as well as TyG index with the severity and mortality of severe coronavirus disease 2019 (COVID-19). A total of 1228 confirmed COVID-19 patients were included in the current research. Regression models were performed to evaluate the correlation between the lipid index and severity and mortality of COVID-19. The TyG index and TG/HDL-C levels were significantly higher in the severe patients (P<0.05). TG/HDL-C, LDL-C/HDL-C, TC/HDL-C ratios, and TyG index were significantly lower in survivor cases (P<0.05). Multivariate logistic regression analysis demonstrated that predictors of the severity adjusted for age, sex and BMI were TyG index, TG/HDL-C ratio (OR = 1.42 CI:1.10–1.82, OR = 1.06 CI: 1.02–1.11, respectively). This analysis showed that TG/HDL-C, TC/HDL-C, LDL-C/HDL-C ratios, and TyG index statistically are correlated with COVID-19 mortality (OR = 1.12 CI:1.06–1.18, OR = 1.24 CI:1.05–1.48, OR = 1.47 CI:1.19–1.80, OR = 1.52 CI:1.01–2.31, respectively). In summary, the TyG index and lipid ratios such as TC/HDL-C, TG/HDL-C, LDL-C/HDL-C could be used as an early indicator of COVID-19 mortality. Furthermore, the study revealed that TyG index and TG/HDL-C indices are biochemical markers of COVID-19 severe prognosis.

## Introduction

In December 2019, a novel coronavirus (2019-nCoV) was discovered from individuals with pneumonia in Wuhan, China [1]. According to the Iranian official reports, the coronavirus disease 2019 (COVID-19) epidemic was started from 2020-02-19 in Iran [2]. The most important characteristic of the COVID-19 pandemic was the rapid spread and increasing trend of the crisis so that this pandemic has affected almost every country in the world quickly. By December 29, 2020, a total of 6,190,000 patients with COVID-19 have been diagnosed in Iran of which 131,000 deaths have been occurred by the virus. Iran ranks tenth in the number of deaths due to COVID- 19 in the world.

Patients with COVID-19 display a wide spectrum of manifestations from mild symptoms and good prognosis to severe lethal respiratory infection [3, 4]. Severe cases of COVID-19 develop acute respiratory distress syndrome and often need mechanical ventilation. So, to limit the increase of severe cases, it seems essential to recognize the factors that promote the development of COVID-19 severity [5]. Although, the severity of COVID-19 is probably to be multifactorial, accumulating evidence has shown a high risk of poor prognosis and more severe complications among peoples with COVID-19 who have comorbidities [6, 7] such as metabolic syndrome, hypertension, cardiovascular disease (CVD), and type 2 diabetes mellites (T2DM) [7]. Indeed, all of these comorbidities are accompanied by insulin resistance (IR) [7]. The IR is caused by defects in insulin action in its target tissues either due to insulin receptor defects or disorders in the post-receptor insulin signaling cascade [8]. It is worse nothing that insulin plays a vital role in normal lung function as well as the management of people with COVID-19 [9]. It is believed that disturbance in metabolic health especially IR is the key risk factor for severe COVID-19 [9–14]. So, early diagnosis of IR, as an important risk factor for poor prognosis of COVID-19, plays a crucial role in predicting the severity and managing the disease. Although most clinicians are conscious of the importance of IR, it is never formed in the routine clinical assessment of COVID-19 patients and causes it challenging to determine the effectiveness of IR in predicting COVID19 outcomes [14]. It is likely due to the time-consuming and expensiveness of the gold standard method of IR assessment, Hyperinsulinaemic-euglycaemic clamp technique [15], and also, lacking standardization, availability, and cost-effective of the insulin assessment method for indirect estimation of IR such as homeostasis model assessment for insulin resistance (HOMA-IR), the fasting glucose to insulin ratio (FG-IR), and the quantitative insulin sensitivity check index (QUICKI) [16]. Therefore, it seems reasonable using simple, inexpensive and more available biochemical indices for IR estimation can be helpful for determining COVID-19 prognosis [14] and can improves therapeutic strategy and disease outcomes at the time of COVID-19 diagnosis [17, 18]. In recent years, some simple and reliable biochemical markers including triglyceride (TG)-glucose (TyG) index [19], and the TG to high-density lipoprotein cholesterol (TG/HDL-C) ratio [20], the low-density lipoprotein cholesterol to HDL-C (LDL-C/HDL-C), and the total cholesterol to HDL-C (TC/ HDL-C) ratio [21] have been developed for estimating IR [22].

Although some studies showed dyslipidemia and its potential association with the outcome of COVID-19 [23–26], it seems that lipid ratios and TyG index can be considered as a better indicator of COVID-19 severity than blood level of lipid profile alone at the time of COVID-19 diagnosis [27, 28]. Given the insufficient studies regarding the correlation between lipid ratios and TyG index and the critical care outcomes of COVID-19 in the large affected population, we aimed to evaluate the possible association of TG/HDL-C, TC/HDL-C, LDL-C/HDL-C, and TyG indices, as a surrogate of estimating IR, with disease severity in non-vaccinated hospitalized COVID-19 patients at the time of diagnosis in Iranian ethnicity.

## Material and methods

### Study population

The present study employed a cross-sectional design. Since the beginning of the outbreak in Iran, a local registration system was created in Shahroud, Iran, in the administration of Shahroud University of Medical Sciences (SHMU). A comprehensive electronic medical record including epidemiological, demographic, anthropometric, chronic medical histories, clinical, and laboratory data was created, from people admitted to SHMU hospitals due to a SARS-CoV-2 infection within February 20, 2020, and March 20, 2021. Only data from hospitalized and non-vaccinated cases with a COVID-19 diagnosis confirmed by real-time reverse transcriptase-polymerase chain reaction (RT-PCR) from oro- and nasopharyngeal swab specimens were included in our analysis. Patients who lacked sufficient data were excluded from the study. Finally, a total of 1228 cases were incorporated in the analysis (S1 Fig). This research was approved by the Ethics Committee of Shahroud University of Medical Sciences (IR. SHMU- REC.1398.160) and was conducted in accordance with the declaration of Helsinki. Informed consent was received from all the cases before registration in the study.

### Clinical and laboratory assessments

Body mass index (BMI) was determined via standard calculation [body weight (kg)/height squared (m2)]. Blood specimens were taken from patients who had been fasting ten-hour at the baseline (COVID-19 diagnosis). Biochemical markers such as fasting blood glucose (FBG), TC, TG, LDL-C, HDL-C, C-reactive protein (CRP) and lactate dehydrogenase (LDH), and ferritin were assessed using calorimetric methods via the commercially available kits (Pars Azmoon, Tehran, Iran). The white blood cell (WBC) and lymphocyte count were also performed. The TG/HDL-C, TC/HDL-C, LDL-C/HDL-C and TyG indices were calculated via the following formulas respectively: TG (mg/dL)/HDL-C (mg/dL), TC (mg/dL)/HDL-C (mg/ dL), LDL-C (mg/dL)/HDL-C (mg/ dL), and Ln [TG (mg/dL) × FBG (mg/dL)]/2.

People with COVID-19 are considered to have the severe disease if they have: 1. respiratory rate>30/min, 2. oxygen saturation≤93%, 3. Patients with shock, or respiratory failure requiring mechanical ventilation, or with other organ failure admission to intensive care unit (ICU). The deceased cases were also considered as severe patients.

### Statistical analysis

Statistical analysis was performed using SPSS software version 23. The chi-squared, Fisher exact test, and Student’s t-test were used to compare differences between two groups. The cut-off values were assessed according to the receiver operating characteristic (ROC) curves and a minimum sensitivity of 50%. univariate and multivariate logistic regression analyses adjusted to age, sex, and BMI also were used. Statistically significant differences were considered with p-values <0.05.

## Results

A total of 1228 confirmed patients were incorporated in the current study, with a mean age of 58.8 ± 16.2 years old. 611 (49.8%) of patients were males. Demographic and clinical features are exhibited in Table 1. Regarding the history of comorbidies, 23.7% had CVD, 24.7% had T2DM, and 6.2% had chronic kidney disease. The most common comorbidities were CVD (24.7%) and T2DM (23.7%) so that they were 2 and 1.3 times higher in severe cases than mild cases, respectively. Also, severe patients had a significantly lower saturated rate of O_2_. Moreover, deceased patients had a higher value of heart rate and a lower saturated rate of O_2_ (All P< 0.05) (Table 1).

**Table 1:** The basic features of patients with COVID-19 in accordance with the severity and mortality.

Variables	Mild (n = 945)	Severe (n = 283)	P-value	Survivor (n = 1140)	Deceased (n = 88)	P-value
**Demographic**						
Gender Male	455(48.1)	156(55.1)	**0.023**	558(48.9)	53(60.2)	**0.041**
Female	490(51.9)	127(44.9)		582(51.1)	35(39.8)	
Age(years)	56.1±0.51	67.3±0.87	**<0.001**	57.68(1.332)	71.52(0.47)	**<0.001**
**Comorbidities**						
T2DM	216(22.9)	87(30.7)	**0.007**	275(24.1)	28(31.8)	0.107
CVD	185(19.6)	106(37.5)	**<0.001**	254(22.3)	37(42)	**<0.001**
Cancer	10(1.1)	11(3.9)	**0.003**	16(1.4)	5(5.7)	**0.014**
COPD	6(0.6)	4(1.4)	0.252	8(0.7)	2(2.3)	0.157
Asthma	24(2.5)	16(5.7)	**0.010**	80(2.8)	8(9.1)	**0.006**
Chronic Liver Disease	16(1.7)	4(1.4)	1.000	20(1.8)	0(0)	0.391
Chronic Kidney Disease	47(5)	29(10.2)	**0.001**	65(5.7)	11(12.5)	**0.011**
Neurological Diseases	28(3.0)	20(7.1)	**0.002**	39(3.4)	9(10.2)	**0.005**
Seizures	7 (0.7)	1(0.4)	0.690	7(0.6)	1(1.1)	**0.041**
**Clinical finding**						
Respiratory rate	12.53±0.79	13.16±0.81	0.582	15.72±1.86	12.65±0.59	0.147
Heart rate	81.13± 1.05	84.04± 1.18	0.072	82.13±0.81	89.09±3.53	**0.017**
Systolic blood pressure	115.79±1.27	117.35±1.31	0.396	116.8±0.95	114.7±3.5	0.533
Diastolic blood pressure	74.08±0.86	74.5±0.92	0.743	74.5±0.65	72.29±2.5	0.341
BMI	27.84±0.15	27.92±0.3	0.806	27.89±0.14	27.33±0.52	0.279
SaO 2	95.68±0.15	90.2±0.74	**<0.001**	92.7±0.48	91.4±0.83	**0.014**

Data were expressed as mean ± standard error of mean or number (percent).

Laboratory findings and lipid ratios of cases according to the severity and mortality have been shown in Table 2. Severe and deceased patients showed statistically significant higher values of FBS, neutrophil/lymphocyte ratio, ESR, WBC, CRP, LDH, and ferritin when compared with mild and survivor groups, respectively (p< 0.05). Regarding the lipid profile, TG level did not demonstrate a significant difference between groups. Serum concentrations of TC, HDL-C, and LDL-C were lower in severe and deceased groups in comparison with mild and survivor groups, respectively (p< 0.05). The severe group showed a significant increase in TG/HDL-C ratio and TyG index compared to the mild patients (p < 0.05). Also, all lipid ratios and TyG index were significantly higher in the deceased group than in the survivor group (p < 0.05) (Table 2).

**Table 2:** Laboratory findings, Lipid profile and lipid ratios of patients with COVID-19.

Variables	Mild (n = 945)	Severe (n = 283)	P-value	Survivor (n = 1140)	Deceased (n = 88)	P-value
**FBG (mg/dl)**	129.94±2.28	143.94±4.56	**0.006**	131.37±2.08	159.45±9.65	**0.006**
**TG (mg/dl)**	105.38±1.98	111.8±3.65	0.118	106.65±1.8	109.10±5.7	0.713
**TC (mg/dL)**	134.13±1.16	127.81±2.61	**0.028**	133.4±1.1	123.92±4.75	**0.03**
**HDL-C (mg/dL)**	33.59±0.32	31.70±0.63	**0.008**	33.53±0.3	28.25±1.12	**<0.001**
**LDL-C (mg/dL)**	76.11±0.72	70.39±1.42	**<0.001**	75.24±0.66	69.05±2.72	**0.014**
**Tg/HDL-C**	3.61±0.11	4.08±0.19	**0.039**	3.63±0.09	4.83±0.46	**0.012**
**TC/HDL-C**	4.24±0.05	4.24±0.09	0.976	4.21±0.04	4.65±0.20	**0.038**
**LDL-c/HDL-C**	2.43±0.03	2.41±0.06	0.817	2.40±0.03	2.75±0.15	**0.021**
**TyG**	8.63±0.023	8.78±0.05	**0.008**	8.65±0.022	8.83±0.1	0.101
**ESR**	34.25±0.82	40.71±2.1	**0.005**	34.99±0.8	45.1±4	**0.016**
**WBC (× 10^9^/L**	32.75±0.54	45.82±1.94	**<0.001**	34.45±0.59	52.16±3.9	**<0.001**
**Lymphocytes (x10e^3^/mL)**	26.044±0.37	22.04±0.75	**<0.001**	25.5±0.35	20.14±1.4	**<0.001**
**NLR**	3.73±0.1	5.62±0.43	**<0.001**	4.02±0.13	6.04±0.56	**0.001**
**CRP (mg/dL)**	25.55±0.92	33.37±1.9	**<0.001**	26.77±0.87	34.16±3.24	**0.025**
**Lactate dehydrogenase, U/L LDH**	453.55±6.8	598.82±18.1	**<0.001**	471.03±6.86	689.78±30.11	**<0.001**
**Ferritin**	292.94±18.3	454.05±37.14	**<0.001**	318.54±17.6	478.84±53.36	**0.012**

Data were expressed as mean ± standard error of mean.

Multivariate logistic regression analysis revealed that predictors of severity adjusted for age, sex and BMI were TyG index, TG/HDL-c ratio, and TG. Notably, TyG was markedly correlated to higher odds of severe disease (OR = 1.42 CI:1.10–1.82). This analysis showed that all lipid ratios and TyG index statistically are correlated with COVID-19 mortality (Table 3).

**Table 3:** Odds ratios for severe and deceased cases associated with lipid profile, TyG index, and lipid ratios.

	Severity			Mortality		
**Variables**	OR(CI)	P-value	AUC	OR(CI)	p-value	AUC
**TG (mg/dl)**	1.004(1.001–1.006)	**0.003**	0.707	1.004(1.001–1.008)	0.069	0.783
**TC (mg/dL)**	0.99(0.99–1.00)	0.586	0.706	0.99(0.98–1.01)	0.493	0.787
**HDL-C (mg/dL)**	0.98(0.97–1.00)	0.054	0.707	0.95(0.92–0.97)	**<0.001**	0.782
**LDL-C (mg/dL)**	0.99(0.98–1.00)	0.062	0.714	0.99(0.98–1.00)	0.415	0.771
**TG/HDL-C**	1.06(1.02–1.11)	**0.007**	0.705	1.12(1.06–1.18)	**<0.001**	0.787
**TC/HDL-C**	1.02(0.91–1.14)	0.684	0.713	1.24(1.05–1.48)	**0.011**	0.794
**LDL-C/HDL-C**	1.03(0.88–1.2)	0.682	0.705	1.47(1.19–1.80)	**<0.001**	0.779
**TyG**	1.42(1.10–1.82)	**0.006**	0.706	1.52(1.01–2.31)	**0.047**	0.781

Regression models adjusted for age sex and BMI.

In addition, the prediction for severity and mortality was also determined by Area Under Curve (AUC). According to AUC, the prediction of lipid ratios and TyG index for death are better than severity. Lipid ratios and TyG index could significantly predict the odds of death more than lipid profile alone. So, increasing each unit in TyG and LDL-C/HDL-C ratio increased the odds of death by 52% and 47% (Table 3).

Cut-off points accordance with a minimum sensitivity of 50% shown in Table 4. Categorical TyG, HDL-C, LDL-C, and their ratio predict the death significantly based on cutoff (Table 4).

**Table 4:** Analysis of the ROC curve of the lipid profile, TyG index, and lipid ratios with mortality in patients with COVID-19.

Variables	Cut off*	Sensitivity	Specificity	P-value**	AUC	Univariate OR(CI)
**TG (mg/dl)**	98.50	50	56.3	0.349	.531	1.28(0.82–1.98)
**TC (mg/dL)**	126.50	50.7	46.5	0.097	.441	0.75(0.48–1.16)
**HDL-C (mg/dL)**	29.50***	54.5	36.8	**<0.001**	.625	**0.48(0.31–0.75)**
**LDL-C (mg/dL)**	65.50***	50	35.8	**0.009**	.583	**0.56(0.36–0.86)**
**TG/HDL-C**	3.22	53.7	57.8	**0.019**	.578	1.27(0.82–1.97)
**TC/HDL-C**	4.24	50.7	57.7	**0.039**	.558	1.13(0.72–1.76)
**LDL-C/HDL-C**	2.44	50	60.4	0.103	.552	**1.66 (1.08–2.58)**
**TyG**	8.77	60.9	63.5	**0.040**	.596	**2.04 (1.31–3.17)**

* Greater than or equal to the cut-off values based on a minimum sensitivity of 50%.

** p-value for Significant differences with 0.5.

***less than or equal to cut off for HDL.

Cut-offs 8.77 and 2.44 for the TyG and LDL-c/HDL-c ratio increased odds of death significantly (OR = 2.04 and 1.66)

## Discussion

Accumulating evidence indicates that loss of metabolic health is a key risk factor for the severity of COVID-19 [9, 29]. It is well characterized that IR and related metabolic disorders especially metabolic syndrome, CVD, and T2DM are correlated with poor prognosis of COVID-19 [30, 31]. In accordance with previous studies [32, 33], the current study also demonstrated that the presence of comorbidities such as T2DM, cancer, asthma, chronic kidney disease, neurological diseases, and CVD are related to more severe illness in COVID-19 patients.

The current study revealed significantly decreased levels of TC, HDL-C, and LDL-C in the severe group and deceased group when compared with the mild group and survivor group respectively. In this regard, meta-analysis researches also demonstrated similar results [34, 35]. It is likely due to the raised usage of cholesterol for pulmonary surfactant synthesis, and/or decreased cholesterol synthesis in the liver due to liver dysfunction in severe cases of COVID-19. Increased pro-inflammatory cytokines may be another reason for these changes in lipid profile through upregulation of the scavenger receptor class B type 1 in COVID-19 infection [35]. While the current study and several other studies established the correlation between lipid profile, biomarkers of metabolic state and disease severity in COVID-19 cases [23, 35, 36], the correlation between IR markers and COVID-19 outcomes has hardly been investigated so far.

It is worth noting that, direct and indirect methods for IR assessment are technically demanding, time-consuming and expensive [18, 37]. Recently, several researchers have suggested that lipid ratios could be valuable alternative biomarkers for estimating IR in several disorders and populations because of their analytical and economic advantages [22, 37–39]. Our result revealed that an increase in TG/HDL-C ratio and TyG index at the time of admission positively correlated with disease severity among Iranian diagnosed people with coronavirus infection after adjusting for BMI, sex, and age. More importantly, we demonstrated for the first time that an increase in TG/HDL-C, TC/HDL-C, LDL-C/HDL-C, and TyG indices, as reliable surrogate markers of IR, positively associated with COVID-19 mortality. Previous researches have revealed that the TyG and lipid ratios such as TC/HDL-C, TG/HDL-C, LDL-C/HDL-C may predict the development of T2DM [40, 41], cancer [12, 42], metabolic syndrome [38, 39]. These indices are predictive markers for the severity of a non-alcoholic fatty liver disease (NAFLD) [43]. TyG is also associated with the severity of cardiovascular outcomes in patients with non-ST and ST-segment elevation myocardial infarction [44, 45]. Moreover, the positive correlation between TG/HDL-C ratio and pulmonary disease is described in asthma, obstructive sleep apnea [46, 47], and idiopathic pulmonary arterial hypertension [48]. It has also been demonstrated that the LDL-C/HDL-C ratio is associated with PaO_2_ levels and severity of pulmonary alveolar proteinosis (PAP). PAP patients had higher TC/HDL-C and TG/HDL-C than the control group [49].

Nevertheless, there is limited information about the importance of lipid ratios in COVID-19. In accordance with our results, Ren et al. revealed that the TyG index is related to a high risk of severity and death in COVID-19 cases [27]. Alcantara-Alonso et al also demonstrated the positive association of TG/HDL-C ratio with LDH, the severity of disease, and the necessity of invasive mechanical ventilation in COVID-19 patients [28]. To our knowledge, the present study is the first study evaluating the association between TC/HDL-C and LDL-C/HDL-C ratios and COVID-19 outcomes. Although our results did not show a significant correlation between TC/HDL-c and LDL-C/HDL-C ratios and disease severity, we revealed that TC/HDL-C and LDL-C/HDL-C indices can predict mortality of COVID-19.

It has been accepted that hyperinsulinemia due to IR promotes SARS-CoV-2 viremia through membrane upregulation of angiotensin-converting enzyme 2 (ACE2) in pneumocytes which in turn is involved in SARS-CoV-2 cell infection [14]. Hyperinsulinemia rises inflammatory markers, impairs fibrinolysis, and increased the risk of coagulation and thrombosis [50]. Although further studies are required to correlate the IR with severity and outcomes of COVID-19, the present study revealed a correlation between IR and COVID-19 severity and mortality through IR estimation. Therefore, it is recommended that consideration can be given to evaluating biochemical markers of IR estimation including TyG index and lipid ratios for prognostic utility in the routine clinical assessment of COVID-19 patients. Also, therapeutic interventions can be used to improve insulin sensitivity and COVID-19 outcomes.

This study is the first comprehensive study with a relatively large sample size that was performed to explore the correlation between TyG index and all lipid ratios including TC/HDL-C, TG/HDL-C, LDL-C/HDL-C with COVID-19 severity and outcomes and to determine the prognostic utility of these markers COVID-19 patient in Iran. However, several limitations in the current study need to be considered. First, lack of data about the lipid profile of the COVID-19 cases before infection. It has been revealed that SARS-CoV-2 can affect metabolic states and lipid profile since the early stages of infection [25]. So, it is not possible to ignore a viral effect on the lipid profile at the time of COVID-19 diagnosis. Second, non-hospitalized patients were not included in our study. Third, direct or indirect methods of IR assessing were not carried out to evaluate the correlation between IR and TyG and lipid ratios indices among our study population.

## Conclusion

In summary, our results showed that the TyG and lipid ratios such as TC/HDL-C, TG/HDL-C, LDL-C/HDL-C were remarkably high in deceased COVID-19 cases. Furthermore, the present study revealed that TyG and TG/HDL-C indices are biochemical indicators of severe prognosis in COVID-19 patients. Our finding also highlighted the high risk of critical care outcomes among COVID-19 cases with insulin resistance.

## Supporting information

S1 Fig Flow chart for patients’ enrollment. (PNG)
