# Supplementary figures and images for: Are lipid ratios and triglyceride-glucose index associated with critical care outcomes in COVID-19 patients?
Source: PLoS One. 2022 Aug 1;17(8):e0272000. doi: 10.1371/journal.pone.0272000 (PMC9342722; doi:10.1371/journal.pone.0272000)

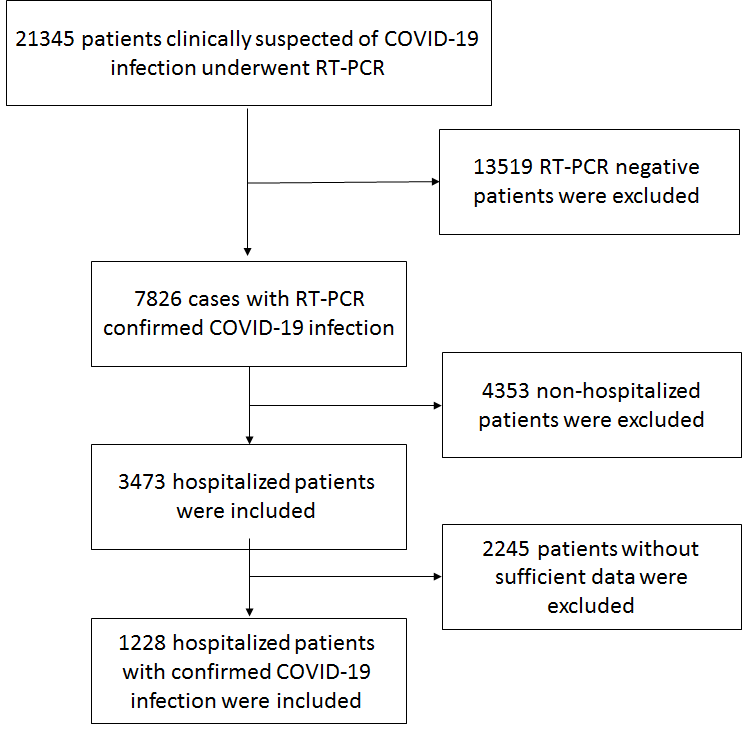

Supplement: S1 Fig — (PNG) [file pone.0272000.s001.png]
